# The Effectiveness of a Computer-Tailored Web-Based Physical Activity Intervention Using Fitbit Activity Trackers in Older Adults (Active for Life): Randomized Controlled Trial

**DOI:** 10.2196/31352

**Published:** 2022-05-12

**Authors:** Stephanie J Alley, Jannique van Uffelen, Stephanie Schoeppe, Lynne Parkinson, Susan Hunt, Deborah Power, Natasha Waterman, Courtney Waterman, Quyen G To, Mitch J Duncan, Anthony Schneiders, Corneel Vandelanotte

**Affiliations:** 1 Physical Activity Research Group Appleton Institute Central Queensland University Rockhampton Australia; 2 Department of Movement Sciences KU Leuven Leuven Belgium; 3 School of Medicine & Public Health College of Health, Medicine, and Wellbeing The University of Newcastle Callaghan Australia; 4 School of Nursing, Midwifery and Social Sciences Central Queensland University Melbourne Australia; 5 School of Health, Medical and Applied Sciences Central Queensland University Bundaberg Australia

**Keywords:** internet, online, activity trackers, activity monitors, wearables, physical activity, mobile phone

## Abstract

**Background:**

Physical activity is an integral part of healthy aging; yet, most adults aged ≥65 years are not sufficiently active. Preliminary evidence suggests that web-based interventions with computer-tailored advice and Fitbit activity trackers may be well suited for older adults.

**Objective:**

The aim of this study was to examine the effectiveness of *Active for Life*, a 12-week web-based physical activity intervention with 6 web-based modules of computer-tailored advice to increase physical activity in older Australians.

**Methods:**

Participants were recruited both through the web and offline and were randomly assigned to 1 of 3 trial arms: tailoring+Fitbit, tailoring only, or a wait-list control. The computer-tailored advice was based on either participants’ Fitbit data (tailoring+Fitbit participants) or self-reported physical activity (tailoring-only participants). The main outcome was change in wrist-worn accelerometer (ActiGraph GT9X)–measured moderate to vigorous physical activity (MVPA) from baseline to after the intervention (week 12). The secondary outcomes were change in self-reported physical activity measured by means of the Active Australia Survey at the midintervention point (6 weeks), after the intervention (week 12), and at follow-up (week 24). Participants had a face-to-face meeting at baseline for a demonstration of the intervention and at baseline and week 12 to return the accelerometers. Generalized linear mixed model analyses were conducted with a *γ* distribution and log link to compare MVPA and self-reported physical activity changes over time within each trial arm and between each of the trial arms.

**Results:**

A total of 243 participants were randomly assigned to tailoring+Fitbit (n=78, 32.1%), tailoring only (n=96, 39.5%), and wait-list control (n=69, 28.4%). Attrition was 28.8% (70/243) at 6 weeks, 31.7% (77/243) at 12 weeks, and 35.4% (86/243) at 24 weeks. No significant overall time by group interaction was observed for MVPA (*P*=.05). There were no significant within-group changes for MVPA over time in the tailoring+Fitbit group (+3%, 95% CI –24% to 40%) or the tailoring-only group (–4%, 95% CI –24% to 30%); however, a significant decline was seen in the control group (–35%, 95% CI –52% to –11%). The tailoring+Fitbit group participants increased their MVPA 59% (95% CI 6%-138%) more than those in the control group. A significant time by group interaction was observed for self-reported physical activity (*P*=.02). All groups increased their self-reported physical activity from baseline to week 6, week 12, and week 24, and this increase was greater in the tailoring+Fitbit group than in the control group at 6 weeks (+61%, 95% CI 11%-133%).

**Conclusions:**

A computer-tailored physical activity intervention with Fitbit integration resulted in improved MVPA outcomes in comparison with a control group in older adults.

**Trial Registration:**

Australian New Zealand Clinical Trials Registry ACTRN12618000646246; https://anzctr.org.au/Trial/Registration/TrialReview.aspx?ACTRN=12618000646246

## Introduction

### Background

Physical activity is important for healthy aging. Physical activity improves health and well-being and reduces the risk of chronic disease [1,2]. Older adults who are physically active have improved mobility, a reduced risk of falls, and a reduced risk of cognitive decline [1]. However, <30% of older adults are meeting the physical activity recommendations of 30 minutes of at least moderate-intensity physical activity on most days [3]. These low levels of physical activity are contributing to Australia’s rising health costs from the aging population [4-6]. Therefore, population-based physical activity interventions with a wide reach are required.

Web-based physical activity interventions are effective in young and middle-aged adults [7], and they may be well suited to older adults (aged ≥65 years). The percentage of older adults using the internet is steadily growing. In 2016, 79% of older Australians were already connected, of whom 85% used the internet daily [8]. Reviews have found web-based physical activity interventions to be effective in older adults [9-11]. However, many of the included studies used existing interventions created for middle-aged adults rather than new interventions specifically developed for older adults [12,13] and included participants as young as 50 years of age [14,15].

Tailored web-based interventions that provide automated personalized physical activity advice based on participants’ characteristics, physical activity, motivation, and environment are effective and may be particularly suited to older adults [16]. This is because older adults have greater diversity of health-related characteristics [3] and because they have expressed the need for physical activity advice to be tailored specifically to them [17]. This expressed need is in line with findings from Ammann et al [18], who found that a tailored web-based physical activity intervention was more effective in older participants than in younger ones. However, very few studies have tested the effectiveness of web-based computer-tailored physical activity interventions created for adults aged ≥65 years and those that have done so demonstrated mixed results [9,11,19,20].

Tailored web-based interventions typically provide participants with personalized advice based on self-reported physical activity data; as such, it is possible that inaccurate advice is delivered because of overreporting of physical activity and social desirability bias [21]. However, commercial activity trackers (eg, Fitbit) allow tailored advice to be based on objectively measured physical activity [22]. A study conducted by Vandelanotte et al [22] found physical activity advice based on Fitbit data to be more credible and lead to greater physical activity changes than advice based on self-reported physical activity data in middle-aged adults. Although older adults do not use activity trackers as frequently as younger adults [23], use is growing steadily in this population [24]. Moreover, multiple studies have found face-to-face, telephone, SMS text messaging, and email advice based on activity tracker data to be effective in older adults [9,25]. Most of these interventions were conducted in older adults with a specific chronic illness (patients with cardiac conditions, chronic obstructive pulmonary disease, or osteoarthritis) and gave feedback on walking and step counts [25]. A recent trial in older adults with no chronic illnesses found a combined web and face-to-face intervention based on Fitbit data to be effective [26]. However, no studies in older adults have investigated the effectiveness of fully automated computer-tailored physical activity advice based on physical activity behavior recorded through a Fitbit activity tracker [27].

### Objectives

The primary aim of the study was to test the effectiveness of a web-based computer-tailored physical activity intervention with Fitbit (Google LLC) integration at increasing objectively measured moderate to vigorous physical activity (MVPA) from before to after the intervention compared with a web-based computer-tailored physical activity intervention without Fitbit integration and a control group in adults aged ≥65 years. The secondary aims were to compare the web-based computer-tailored intervention with and without Fitbit integration and the control group on objectively measured sedentary behavior from before to after the intervention and to compare subjectively measured physical activity and sitting time changes at the midintervention point, after the intervention, and at follow-up.

We hypothesized that the web-based computer-tailored physical activity intervention with Fitbit integration would lead to increased objectively measured MVPA and self-reported physical activity over time and decreased objectively measured sedentary behavior and self-reported sitting time over time compared with the web-based computer-tailored physical activity intervention without Fitbit integration and a control group.

## Methods

### Study Design

A 3-arm randomized controlled trial was conducted where participants were randomized into one of three groups: (1) tailoring+Fitbit, (2) tailored advice only, and (3) wait-list control. Participants completed web-based surveys at baseline (week 0), at the midintervention point (week 6), after the intervention (week 12), and at follow-up (week 24). Objective physical activity and sedentary behavior were collected by means of wrist-worn accelerometry at baseline and week 12. More detail of the methods can be found in a protocol paper of the trial [28].

### Participants

Participants were recruited in Rockhampton (regional Queensland), Bundaberg (regional Queensland), and Adelaide (metropolitan South Australia), Australia, through paid Facebook advertising, email lists, flyers, and local newsletters. Recruitment was carried out between April 2018 and March 2019, and data collection was completed in November 2019. Eligible participants were English-speaking adults aged ≥65 years who had internet access and basic internet confidence, could attend 2 face-to-face appointments at one of the project locations, and could safely increase their physical activity as determined by the Physical Activity Readiness Questionnaire [29] or general physician approval. Eligible participants were those not meeting the physical activity guidelines [30], as assessed by asking participants the following question: “Are you currently participating in less than 30 minutes of physical activity on 5 days a week?” Participants were ineligible if they were already participating in another physical activity program or had used a Fitbit activity tracker in the previous 6 months.

### Sample Size Analysis

To detect differences between the 2 intervention groups and the control group for accelerometer-measured MVPA from baseline to after the intervention, 100 participants per group were required. This was to detect an effect size of 0.37 based on the average effect size of web-based physical activity interventions for inactive adults [31]. This accounted for a dropout rate of up to 30%. Power was set at 0.80 and the *α* at .05. The decision to end participant recruitment was made by the lead (SJA) and senior (CV) investigators because the trial was close to the sample size goal (n=243), the remaining funds were limited, and interest in the trial had slowed.

### Procedures

Advertising materials directed prospective participants to the landing page of the intervention website, which had more details about study participation and access to the participant information sheet and eligibility survey. Prospective participants were automatically notified of their eligibility upon completion of the survey, and eligible participants received a welcome email. Participants were asked to complete web-based research surveys at baseline, week 6, week 12, and week 24 through the intervention website. Participants indicated their informed consent through a check box at the beginning of the baseline survey. If participants missed a survey, they were still asked to complete later surveys. Participants were posted an accelerometer to wear on their wrist for 7 consecutive 24-hour days, including when sleeping and showering, at baseline and week 12. The blinded accelerometers were only used for research evaluation and were not part of the intervention. Participants attended a baseline appointment to return the baseline accelerometer and were randomly allocated to one of the three trial arms (tailoring+Fitbit, tailored advice only, and wait-list control). Randomization lists were created by the lead investigator (SJA) using computer-automated block randomization with block sizes of 15 and a 1:1:1 ratio. Randomization was stratified by sex (male and female) and age (<75 years and >75 years) to ensure an equal distribution of men and women in different age groups over the intervention arms. Using the randomization lists, the research manager (DP) and research assistants (CW and NW) assigned the participants by date of baseline appointment. Because of the nature of the intervention, neither the researchers nor the participants were blinded to group allocation. During the appointment, after randomization, intervention group participants were shown through the *Active for Life* intervention website and Fitbit participants were provided with a Fitbit activity tracker and shown how to sync it to the intervention website. After the 12-week intervention, participants attended another face-to-face follow-up appointment to return the week 12 accelerometer. Participants received up to 3 email reminders for each research survey. If the surveys were still incomplete after the reminders, participants were offered a voucher worth Aus $20 (US $15) to complete them within the next few days. Participants received a voucher worth Aus $50 (US $37) after completing all research surveys. Wait-list participants were given access to the intervention after completing the week 24 research survey.

### Intervention

**Figure 1:**
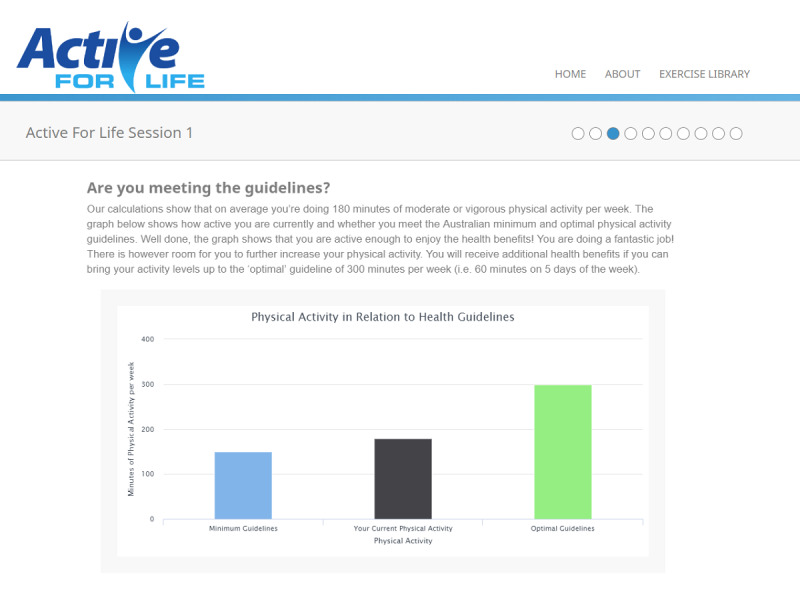
Tailored advice sample.

An in-depth description of the intervention can be found elsewhere [28]. The *Active for Life* intervention is a 12-week web-based program with 6 modules of tailored advice delivered biweekly. The modules of tailored advice are computer automated and use participant data to select appropriate messages from a database of messages using if-then algorithms (eg, if *low self-efficacy and inactive* then *message on improving self-efficacy by starting small*). The advice is based on the theory of planned behavior [32] and the social cognitive theory [33] and includes evidence-based behavior change techniques [34,35]. The advice encourages participants to work toward meeting the physical recommendations of 30 minutes of moderate-intensity physical activity on at least 5 days each week, including 2 to 3 sessions of strength and flexibility activity. Participants were also encouraged to limit their sitting time to <8 hours per day and to take regular breaks from sitting. Specifically, the advice covers the physical activity recommendations, physical activity benefits, safety when exercising, exercising with a chronic disease, sedentary behavior, goal setting, action plans, self-efficacy, physical activity barriers, social and physical environments, rewards, habit formation, and relapse prevention. The physical activity advice is tailored to participants’ characteristics and environment, physical activity behavior, and psychosocial correlates of physical activity (eg, self-efficacy and social support). Each module of advice included approximately 10 sections that participants scroll through, each with a paragraph on a new subtopic (eg, *Are you meeting the guidelines?*, *Losing weight*, and *Exercise with arthritis*). Some sections include a graph or a picture (Figure 1).

The intervention website includes an action-planning tool that participants are encouraged to complete at the end of modules 2 and 4. The tool guides participants in setting an action plan (what, where, when, and with whom) for being active in the following fortnight. The intervention website also includes an exercise library where participants can access strength and flexibility exercise plans, written by a physiotherapist, suitable for the beginner and intermediate levels. The plans are 4 weeks in duration, and participants can view videos of the exercises through a link to an external website (Physitrack PLC).

Both the tailoring+Fitbit and the tailoring-only participants were given access to the same intervention, including the 6 modules of computer-tailored advice, action-planning tool, and exercise library. Both groups completed a brief questionnaire at the start of each module to inform the computer-tailored advice. The only difference was that the tailoring+Fitbit participants were required to sync their Fitbit activity tracker with the website to measure their physical activity over the past 2 weeks, whereas the tailoring-only group answered a few additional questions that asked them to recall how many minutes of physical activity they had completed in the past 2 weeks. All other questions (eg, sitting time, self-efficacy, and social support) were identical. Participants in the control group initially only received access to the research surveys, but after completing the week 24 survey, they also received access to the tailored advice modules, action-planning tool, and exercise library.

### Measures

Objective measurement of physical activity and sedentary behavior was carried out by means of wrist-worn ActiGraph GT9X accelerometers at baseline and week 12. Accelerations were recorded at 30 Hz. Accelerometer data were processed through ActiLife and valid wear time was set at 16 hours each day on a minimum of 5 days. Nonwear time was assessed based on vector magnitude using the Choi et al [36] algorithm. This algorithm defines nonwear time as 90 consecutive minutes of 0 counts per minute, with 2-minute interruptions allowed. Physical activity behavior was defined using vector-magnitude cut points for older adults as determined by Kamada et al [37]. Sedentary behavior was defined as <2000 counts per minute, light physical activity as 2000 to 8249 counts per minute, and MVPA as ≥8250 counts per minute. Periods of sleep were determined by the Tudor-Locke algorithm [38].

Physical activity was also assessed in all groups at baseline, week 6, week 12, and week 24 by means of the Active Australia Survey [39], which measures time spent walking and in MVPA over the previous week. Total weekly physical activity is calculated by adding time spent walking, time spent in moderate physical activity, and time spent in vigorous activity doubled to account for the extra energy expenditure. The Active Australia Survey is reliable (intraclass correlation coefficient=0.64) [40] and validated compared with accelerometer-derived MVPA (*r*=0.35) in older adults [41].

Sitting time was assessed at baseline, week 6, week 12, and week 24 by means of the Workforce Sitting Questionnaire [42]. The questionnaire measures minutes of sitting time per week during work, television viewing, computer use outside work, transport, and other leisure-time activities on work and nonwork days. Daily sitting time on work and nonwork days is calculated by adding sitting time during all activities on work and nonwork days, respectively. Weekly sitting time is calculated by multiplying sitting time on work and nonwork days by the number of days worked and not worked, respectively, and then adding both outcomes. Average daily sitting time is calculated by dividing weekly sitting time by 7. The Workforce Sitting Questionnaire has adequate test-retest reliability (intraclass correlation coefficient=0.46-0.90) and validity compared with accelerometery (women: *r*=0.22-0.46, men: *r*=0.18-0.29) for both work and nonwork days [42].

Participant demographics, including sex, age, marital status (single or married or de facto relationship), height and weight (to calculate BMI), English as main language (yes or no), education level (primary, secondary, technical college, or university), employment (full time, part time, or not working), pretax household income (<Aus $41,599 [US $30,658], Aus $41,600 [US $30,659] to Aus $64,999 [US $47,904], Aus $65,000 [US $47,905] to Aus $103,999 [US $76,647], or ≥Aus $104,000 [US $76,648]), and current health diagnosis (yes or no) were measured at baseline. Internet self-efficacy as assessed by means of the valid and reliable Internet Self-efficacy Scale was also assessed at baseline [43]. The Internet Self-efficacy Scale includes 8 items of internet skills on a 7-point Likert scale ranging from 7 (*strongly agree*) to 1 (*strongly disagree*). Items are added together to produce a summary score; higher scores indicate higher internet self-efficacy.

### Data Analysis

Analyses of primary and secondary outcomes followed the intention-to-treat principle. Separate generalized linear mixed model analyses were conducted to test the primary outcome of changes in accelerometer-measured MVPA by group and to test the secondary outcomes of changes in self-reported physical activity, accelerometer-measured sedentary behavior, and self-reported sitting time by group. A *γ* distribution with log link was used for the analyses on accelerometer-measured MVPA and self-reported physical activity because of positively skewed distributions. A normal distribution with identity link was used for the analyses on accelerometer-measured sedentary behavior and self-reported sitting time. Group (tailoring+Fitbit, tailoring only, and wait-list control) by time (baseline and week 12) interactions on accelerometer-measured MVPA and sedentary behavior were analyzed. These analyses controlled for accelerometer wear time. Group (tailoring+Fitbit, tailoring only, and wait-list control) by time (baseline, week 6, week 12, and week 24) interactions on self-reported physical activity and sitting time were analyzed. A sensitivity analysis was conducted to determine the effect of missing data on analysis outcomes. Under the assumption of missing at random, missing values were imputed through chained equations. The fully conditional specification was used to create 20 imputed data sets that were used to conduct the sensitivity analysis. Analyses were conducted using SAS (version 9.4; SAS Institute Inc) with an *α* of .05.

### Ethics Approval

Ethics approval was received from the Central Queensland University Human Ethics Committee before data collection commenced (H16/12-321).

## Results

### Overview

**Figure 2:**
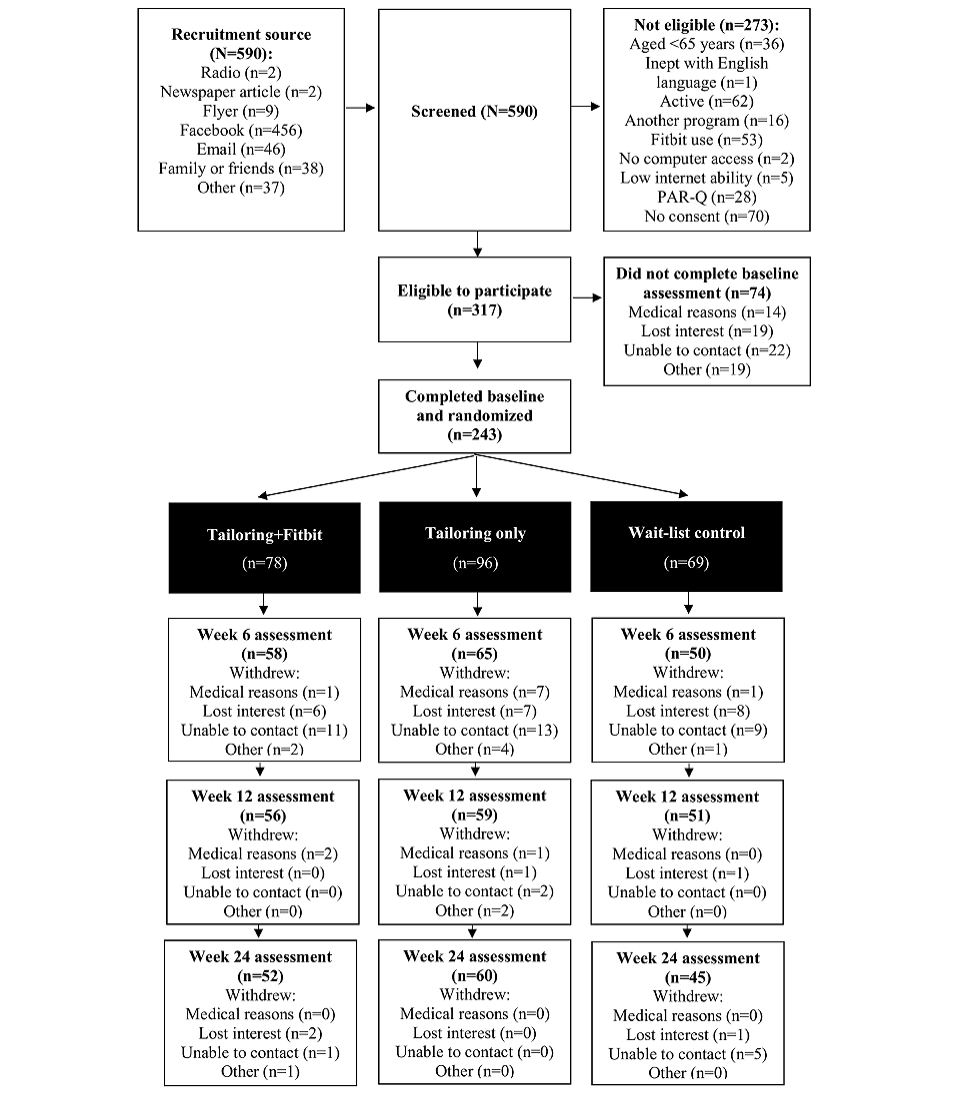
Flowchart of study participants.

Figure 2 shows the flow of participants through the trial. Of the 590 participants screened, 317 (53.7%) met the eligibility criteria and 243 (41.2%) completed their baseline assessment and were randomized. Attrition was 28.8% (70/243) at 6 weeks, 31.7% (77/243) at 12 weeks, and 35.4% (86/243) at 24 weeks.

Table 1 shows baseline participant characteristics. Among the 243 participants, 191 (78.6%) were women; 151 (62.1%) were from Adelaide, South Australia; 231 (95.1%) spoke English as their primary language; 172 (70.8%) were married; 126 (51.9%) had a university education; 178 (73.3%) were not working; 89 (36.6%) had a chronic disease; 184 (75.7%) used the internet several times a day; and 84 (44.4%) had a household income <Aus $40,000 (US $29,595). The average age was 69 (SD 4.32; range 65-98) years. The average BMI was 30 (SD 28.84) kg/m^2^ (overweight), and the average internet self-efficacy was good at 44 (SD 47.00) out of 56 [43]. Participants who completed the week 12 outcomes had a lower baseline BMI (mean 28.95, SD 5.91, kg/m^2^) than participants who did not (mean 31.41, SD 6.37, kg/m^2^). No other differences were observed for demographics, health, or internet use between participants who completed the week 12 outcomes and those who did not.

**Table 1:** Baseline participant characteristics (N=243).

Baseline characteristics			All participants	Tailoring+Fitbit (n=78)	Tailoring only (n=96)	Wait-list control (n=76)	Dropout (n=77)		Completer (n=166)	Chi-square (*df*)		*t* test (*df*)		*P* value	
**Sex, n (%)**											0.3 (241)		N/A^a^		.62
	Male		52 (21.4)	18 (23.1)	19 (19.8)	15 (21.7)	24 (46.2)		28 (53.8)						
	Female		191 (78.6)	60 (76.9)	77 (80.2)	54 (78.3)	78 (40.8)		113 (59.2)						
**Location, n (%)**											0.6 (241)		N/A		.76
	Rockhampton, Queensland		74 (30.5)	24 (30.8)	36 (37.5)	14 (20.3)	24 (32.4)		50 (67.6)						
	Bundaberg, Queensland		18 (7.4)	8 (10.3)	5 (5.2)	5 (7.2)	7 (38.9)		11 (61.1)						
	Adelaide, South Australia		151 (62.1)	46 (59)	55 (57.3)	50 (72.5)	46 (30.5)		105 (69.5)						
**Primary language, n (%)**											0.6 (241)		N/A		.53
	English		231 (95.1)	72 (92.3)	94 (97.9)	65 (94.2)	72 (31.2)		159 (68.8)						
	Other		12 (4.9)	6 (7.7)	2 (2.1)	4 (5.8)	5 (41.7)		7 (58.3)						
**Marital status, n (%)**											0.02 (241)		N/A		.99
	Single		71 (29.2)	22 (28.2)	32 (33.3)	17 (24.6)	22 (31)		49 (69)						
	Married or de facto relationship		172 (70.8)	56 (71.8)	64 (66.7)	52 (75.4)	55 (32)		117 (68)						
**Education, n (%)**											1.8 (241)		N/A		.41
		Secondary school	61 (25.1)	25 (32.1)	21 (21.8)	15 (21.7)	19 (31.1)		42 (68.9)						
		Technical college	56 (23)	11 (14.1)	29 (29.2)	17 (24.6)	14 (25)		42 (75)						
		University	126 (51.9)	42 (53.8)	47 (49)	37 (53.6)	44 (34.9)		82 (65.1)						
**Employment, n (%)**											2.6 (241)		N/A		.28
	Full time		22 (9.1)	8 (10.3)	7 (7.3)	7 (10.1)	7 (31.8)		15 (68.2)						
	Part time or casual		43 (17.7)	16 (20.5)	15 (15.7)	12 (17.3)	18 (41.9)		25 (58.1)						
	Not working		178 (73.3)	54 (69.2)	74 (77.1)	50 (72.5)	52 (29.2)		126 (70.8)						
**Chronic disease status, n (%)**											1.2 (241)		N/A		.32
	Yes		89 (36.6)	26 (33.3)	35 (36.5)	28 (40.6)	32 (36)		57 (64)						
	No		154 (63.4)	52 (66.7)	61 (63.5)	41 (59.4)	45 (29.2)		109 (70.8)						
**Internet use, n (%)**											1.9 (241)		N/A		.39
	Once to several times a week		25 (10.3)	8 (10.3)	13 (13.5)	4 (5.8)		10 (40)	15 (60)						
	Once a day		34 (14)	6 (7.7)	18 (18.8)	10 (14.5)		8 (23.5)	26 (76.5)						
	Several times a day		184 (75.7)	64 (82.1)	65 (67.7)	55 (79.7)		59 (32.1)	125 (67.9)						
**Income,^b^ Aus $ (US $), n (%)**											2.0 (187)		N/A		.58
	>104,000 (>76,957)		24 (12.7)	9 (11.5)	9 (9.4)	6 (8.7)		6 (25)	18 (75)						
	65,000 to 103,999 (48,098 to 76,957)		29 (15.3)	10 (12.8)	10 (10.4)	9 (13)		8 (27.6)	21 (72.4)						
	41,000 to 64,999 (30,339 to 48,098)		52 (27.5)	11 (14.1)	26 (27.1)	15 (21.7)		19 (36.5)	33 (63.5)						
	<40,000 (<29,599)		84 (44.4)	30 (38.5)	33 (34.4)	21 (30.4)		22 (26.2)	62 (73.8)						
Age (years), mean (SD)			69.34 (4.32)	69.88 (4.10)	69.12 (4.93)	68.84 (3.85)	69.08 (3.77)		69.46 (4.56)	N/A		0.64 (241)		.52	
BMI,^c^ mean (SD)			29.73 (28.84)	29.34 (28.40)	29.46 (28.23)	30.52 (29.74)	31.41 (6.37)		28.95 (5.91)	N/A		2.91 (237)		.004	
Internet self-efficacy, mean (SD)			43.90 (47.00)	43.74 (44.50)	44.92 (52.00)	42.65 (45.00)	43.31 (12.43)		44.17 (12.43)	N/A		0.50 (241)		.62	

^a^N/A: not applicable.

^b^Income missing (did not wish to disclose): n=54.

^c^BMI missing: n=4.

**Figure 3:**
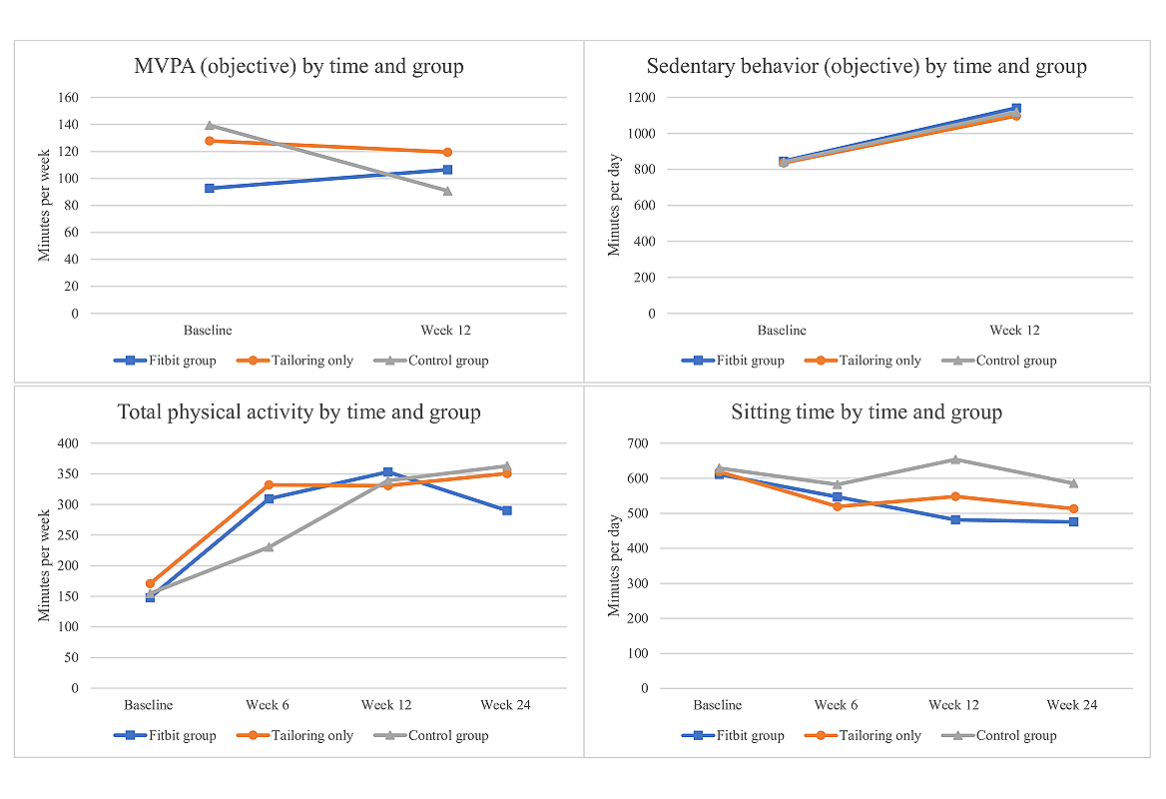
Moderate to vigorous physical activity (MVPA), total physical activity, sedentary behavior, and sitting time by time and group.

Table 2 and Figure 3 present the descriptives for accelerometer-measured MVPA and sedentary behavior and self-reported physical activity and sitting time by time and group. MVPA slightly increased in the tailoring+Fitbit group and decreased in the tailoring-only and control groups, whereas sedentary behavior increased in all groups. Self-reported physical activity more than doubled in all groups, and sitting time decreased in the 2 intervention groups. Sitting time remained relatively constant in the control group.

**Table 2:** Descriptives of physical activity and sedentary behavior by group and time (N=243).

		Tailoring+Fitbit group, mean (SD)	Tailoring only, mean (SD)	Control group, mean (SD)
**Accelerometer-measured moderate to vigorous physical activity minutes per week**				
	Baseline (n=209)	92.75 (84.28)	127.89 (111.30)	139.44 (200.20)
	Week 12 (n=141)	106.54 (127.47)	119.56 (102.13)	90.86 (98.00)
**Self-reported total physical activity minutes per week**				
	Baseline (n=243)	147.95 (152.69)	170.62 (253.26)	154.64 (214.89)
	Week 6 (n=173)	309.31 (202.28)	331.85 (336.85)	230.40 (278.45)
	Week 12 (n=166)	353.21 (309.00)	330.68 (256.45)	339.02 (354.46)
	Week 24 (n=157)	290.19 (268.09)	350.67 (261.73)	362.89 (362.85)
**Accelerometer-measured sedentary minutes per day**				
	Baseline (n=209)	846.70 (242.17)	836.91 (206.95)	842.29 (230.64)
	Week 12 (n=141)	1142.53 (173.58)	1098.43 (142.60)	1119.93 (149.46)
**Self-reported sitting minutes per day**				
	Baseline (n=240)	611.14 (236.47)	618.47 (240.64)	629.04 (232.46)
	Week 6 (n=172)	546.70 (257.83)	519.33 (234.15)	582.11 (273.23)
	Week 12 (n=165)	481.14 (227.27)	547.77 (260.35)	653.70 (247.49)
	Week 24 (n=156)	475.27 (233.65)	513.05 (253.75)	585.30 (252.34)
**Accelerometer wear time in minutes per day**				
	Baseline (n=209)	1199.70 (203.04)	1212.84 (197.44)	1218.25 (190.86)
	Week 12 (n=141)	1440.00 (0.00)	1440.00 (0.00)	1440.00 (0.00)

### Main Analyses and Sensitivity Analyses

Tables 3 and 4 show the changes in accelerometer-measured MVPA, self-reported total physical activity, accelerometer-measured sedentary behavior, and self-reported sitting time by time and group. Results from the main analyses as well as the sensitivity analyses are presented. There was an overall time by group interaction for MVPA, but this was just above the criterion for significance (*P*=.05). No significant improvements in accelerometer-measured MVPA were observed from baseline to after the intervention within any group; however, the control group participants significantly decreased their MVPA by 35%. A pairwise difference was observed between the tailoring+Fitbit group and the control group, where the tailoring+Fitbit group participants increased their MVPA between baseline and week 12 by 59% more than those in the control group. This effect remained in the sensitivity analysis; however, the magnitude was reduced (43% difference). An overall time by group interaction was observed for self-reported total physical activity. All groups increased their self-reported physical activity from baseline to week 6, week 12, and week 24, and pairwise group comparisons revealed that this increase was 61% greater in the tailoring+Fitbit group than in the control group at 6 weeks. The effect was smaller in the sensitivity analysis (34% difference) and did not meet the criterion for significance.

No overall time by group interaction was observed for accelerometer-measured sedentary behavior. The tailoring+Fitbit and control groups significantly increased their accelerometer-measured sedentary behavior from baseline to after the intervention (by 57 and 56 minutes per day, respectively). The magnitude of these effects was lower in the sensitivity analysis (by 34 and 38 minutes per day, respectively) and did not meet the criterion for significance in the tailoring+Fitbit group. No pairwise group differences were observed. No overall time by group interaction was observed for self-reported sitting time. The tailoring+Fitbit group participants decreased their self-reported sitting time between baseline and week 6, 12, and 24 (by 67, 119, and 123 minutes per day, respectively). The tailoring-only group participants decreased their self-reported sitting time between baseline and week 6 and 24 (by 99 and 106 minutes per day, respectively). These effects remained in the sensitivity analysis; however, the magnitudes were reduced. Pairwise group comparisons revealed that the tailoring+Fitbit group participants decreased their sitting time between baseline and after the intervention 133 minutes per day more than those in the control group. However, the magnitude of effect was lower in the sensitivity analysis (98-minute decrease per day) and did not meet the criterion for significance.

**Table 3:** Main analysis, comparison of physical activity by time and group.

			Baseline to 6 weeks, estimate (95% CI)		*P* value		Baseline to 12 weeks, estimate (95% CI)		*P* value		Baseline to 24 weeks, estimate (95% CI)		*P* value		Group×time *P* value	
**Accelerometer-measured moderate to vigorous physical activity per week^a,b^**			—^c^		—		—		—		—		—		.05	
	Tailoring+Fitbit (n=78)	—		—		1.03 (0.76 to 1.40)		.83		—		—		—		
	Tailoring only (n=96)	—		—		0.96 (0.71 to 1.30)		.79		—		—		—		
	Control (n=69)	—		—		0.65 (0.48 to 0.89)		.006		—		—		—		
	Tailoring+Fitbit vs control	—		—		1.59 (1.06 to 2.38)		.02		—		—		—		
	Tailoring only vs control	—		—		1.48 (0.99 to 2.20)		.06		—		—		—		
	Tailoring+Fitbit vs tailoring only	—		—		1.08 (0.73 to 1.59)		.71		—		—		—		
**Self-reported total physical activity per week^a^**			—		—		—		—		—		—		.02	
	Tailoring+Fitbit (n=78)	2.31 (1.83 to 2.93)		<.001		2.24 (1.80 to 2.77)		.001		1.83 (1.44 to 2.32)		<.001		—		
	Tailoring only (n=96)	2.11 (1.64 to 2.72)		<.001		2.19 (1.71 to 2.80)		<.001		2.28 (1.82 to 2.87)		<.001		—		
	Control (n=69)	1.44 (1.07 to 1.93)		.01		1.99 (1.53 to 2.59)		<.001		2.24 (1.61 to 3.12)		<.001		—		
	Tailoring+Fitbit vs control	1.61 (1.11 to 2.33)		.01		1.12 (0.80 to 1.58)		.52		0.82 (0.55 to 1.22)		.32		—		
	Tailoring only vs control	1.47 (1.00 to 2.16)		.05		1.10 (0.77 to 1.58)		.61		1.02 (0.68 to 1.52)		.92		—		
	Tailoring+Fitbit vs tailoring only	1.10 (0.78 to 1.55)		.59		1.02 (0.74 to 1.41)		.90		0.80 (0.58 to 1.11)		.18		—		
**Accelerometer-measured sedentary time per day^b,d^**			—		—		—		—		—		—		.79	
	Tailoring+Fitbit (n=78)	—		—		57.50 (9.65 to 105.34)		.02		—		—		—		
	Tailoring only (n=96)	—		—		38.66 (–6.10 to 83.42)		.09		—		—		—		
	Control (n=69)	—		—		56.07 (6.09 to 106.04)		.03		—		—		—		
	Tailoring+Fitbit vs control	—		—		1.43 (–66.67 to 69.54)		.96		—		—		—		
	Tailoring only vs control	—		—		–17.41 (–81.02 to 46.20)		.59		—		—		—		
	Tailoring+Fitbit vs tailoring only	—		—		18.84 (–42.03 to 79.70)		.55		—		—		—		
**Self-reported sitting time per day^d^**			—		—		—		—		—		—		—	
	Tailoring+Fitbit (n=78)	–67.47 (–129.93 to –5.02)		.03		–119.36 (–190.19 to –48.53)		.001		–123.04 (–201.38 to –44.70)		.002		.13		
	Tailoring only (n=96)	–99.41 (–169.81 to –29.00)		.005		–68.58 (–144.76 to 7.60)		.08		–106.13 (–183.01 to –29.24)		.006		—		
	Control (n=69)	–58.29 (–120.50 to 3.93)		.06		13.68 (–50.66 to 78.01)		.68		–37.44 (–105.7 to 30.83)		.29		—		
	Tailoring+Fitbit vs control	–9.19 (–96.16 to 77.79)		.84		–133.04 (–228.45 to –37.63)		.007		–85.60 (–187.84 to 16.64)		.10		—		
	Tailoring only vs control	–41.12 (134.26 to 52.02)		.38		–82.25 (–181.59 to 17.08)		.11		–68.68 (–171.22 to 33.85)		.19		—		
	Tailoring+Fitbit vs tailoring only	31.93 (–61.69 to 125.56)		.50		–50.78 (–153.91 to 52.34)		.33		–16.91 (–125.93 to 92.10		.76		—		

^a^Reported as percentage change.

^b^Analyses controlled for accelerometer wear time.

^c^Not available.

^d^Reported as mean difference.

**Table 4:** Sensitivity analysis, comparison of physical activity by time and group.

		Baseline to 6 weeks, estimate (95% CI)	*P* value	Baseline to 12 weeks, estimate (95% CI)	*P* value	Baseline to 24 weeks, estimate (95% CI)	*P* value
**Accelerometer-measured moderate to vigorous physical activity per week^a,b^**		—^c^	—	—	—	—	—
	Tailoring+Fitbit (n=78)	—	—	1.14 (0.90 to 1.42)	.26	—	—
	Tailoring only (n=96)	—	—	1.02 (1.01 to 1.06)	.83	—	—
	Control (n=69)	—	—	0.79 (0.60 to 1.05)	.10	—	—
	Tailoring+Fitbit vs control	—	—	1.43 (1.02 to 2.01)	.04	—	—
	Tailoring only vs control	—	—	1.28 (1.23 to 1.35)	.12	—	—
	Tailoring+Fitbit vs tailoring only	—	—	1.12 (1.20 to 1.49)	.47	—	—
**Self-reported total physical activity per week^a^**		—	—	—	—	—	—
	Tailoring+Fitbit (n=78)	2.27 (1.79 to 2.89)	<.001	2.36 (1.90 to 2.92)	<.001	2.08 (1.63 to 2.66)	<.001
	Tailoring only (n=96)	2.10 (1.62 to 2.72)	<.001	2.23 (1.72 to 2.86)	<.001	2.25 (1.77 to 2.86)	<.001
	Control (n=69)	1.68 (1.27 to 2.25)	<.001	2.20 (1.70 to 2.89)	<.001	2.48 (1.84 to 3.35)	<.001
	Tailoring+Fitbit vs control	1.34 (0.92 to 1.93)	.12	1.06 (1.31 to 1.49)	.71	0.84 (0.58 to 1.22)	.36
	Tailoring only vs control	1.23 (0.84 to 1.82)	.28	1.00 (0.69 to 1.45)	.99	0.90 (0.62 to 1.34)	.61
	Tailoring+Fitbit vs tailoring only	1.08 (0.97 to 1.17)	.66	1.06 (0.76 to 1.49)	.71	0.93 (0.67 to 1.28)	.65
**Accelerometer-measured sedentary time per day^b,d^**		—	—	—	—	—	—
	Tailoring+Fitbit (n=78)	—	—	34.49 (–0.33 to 69.31)	.05	—	—
	Tailoring only (n=96)	—	—	24.12 (–2.02 to 50.27)	.07	—	—
	Control (n=69)	—	—	38.09 (4.81 to 71.36)	.02	—	—
	Tailoring+Fitbit vs control	—	—	–3.60 (–52.47 to 45.27)	.88	—	—
	Tailoring only vs control	—	—	–13.96 (–55.92 to 27.99)	.51	—	—
	Tailoring+Fitbit vs tailoring only	—	—	10.36 (–32.88 to 53.61)	.64	—	—
**Self-reported sitting time per day^d^**		—	—	—	—	—	—
	Tailoring+Fitbit (n=78)	–56.95 (–123.61 to –9.72)	.09	–90.39 (–165.56 to –15.22)	.02	–109.73 (–191.90 to –27.56)	.009
	Tailoring only (n=96)	–82.83 (–151.46 to –14.21)	.02	–56.09 (–130.50 to 18.32)	.14	–91.71 (–171.12 to –12.31)	.02
	Control (n=69)	–62.49 (–136.69 to 11.71)	.10	7.57 (–64.09 to 79.24)	.83	–55.75 (–136.57 to 25.07)	.18
	Tailoring+Fitbit vs control	5.54 (–90.15 to 101.24)	.91	–97.97 (–200.78 to 4.85)	.06	–53.97 (–169.52 to 61.57)	.36
	Tailoring only vs control	–20.34 (–116.74 to 76.06)	.68	–63.66 (–161.89 to 34.56)	.20	–35.96 (–148.64 to 76.72)	.53
	Tailoring+Fitbit vs tailoring only	25.89 (–68.65 to 120.43)	.59	–34.30 (–135.56 to 66.96)	.51	–18.01 (–123.33 to 87.30)	.74

^a^Reported as percentage change.

^b^Analyses controlled for accelerometer wear time.

^c^Not available.

^d^Reported as mean difference.

## Discussion

### Principal Findings

The main aim of the study was to determine the effectiveness of a computer-tailored physical activity intervention with Fitbit integration compared with a tailoring-only group and a control group at increasing MVPA from before to after the intervention. The second aim was to determine the effectiveness of a computer-tailored physical activity intervention with Fitbit integration compared with a tailoring-only group and a control group at increasing self-reported physical activity from before the intervention to the midintervention point, after the intervention, and follow-up. The findings showed that there were no significant MVPA changes in the tailoring+Fitbit group or tailoring-only group, whereas there was a decrease in the MVPA of the control group. MVPA increased more in the tailoring+Fitbit group than in the control group. All groups reported increasing their self-reported physical activity, and this increase was greater in the tailoring+Fitbit group than in the control group at the midintervention point. Together, these findings support past studies that have demonstrated that face-to-face, telephone, SMS text messaging, and email physical activity interventions using activity trackers are effective in older adults compared with a control group [25-27]. Most of these past studies focused on self-monitoring of steps and walking, whereas this study provided tailored feedback based on activity tracker–measured light-, moderate-, and vigorous-intensity physical activity. However, it should be noted that the Fitbit device had 5 lights, each of them indicating an additional 2000 steps reached for the day, which may have also motivated this group to maintain their physical activity, independent of the computer-tailored advice. Furthermore, the control group participants had a higher level of MVPA at baseline and therefore had more room to decrease their MVPA. This may have contributed to the between-group difference observed between the tailoring+Fitbit group and the control group on MVPA changes at week 12. Overall, these findings add to the literature by indicating that computer-tailored advice based on Fitbit measurement of light, moderate, and vigorous physical activity is likely to lead to improved physical activity outcomes compared with a control group.

The effectiveness of the tailoring+Fitbit intervention compared with a control group may be further improved by increasing the frequency of the feedback provided. Larsen et al [25] conducted a systematic review on physical activity trackers for older adults and found that only the interventions providing daily feedback on activity tracker data were effective. Our intervention provided in-depth feedback and theory-based behavior change support that would not be feasible to deliver daily. However, daily self-monitoring feedback on minutes of light, moderate, and vigorous physical activity might be feasible, in addition to the biweekly computer-tailored advice. Previous interventions for older adults have successfully delivered basic daily feedback on steps from activity trackers through smartphone apps in graphical and written form [44,45].

This study found no significant difference in accelerometer-measured MVPA or self-reported physical activity in the tailoring+Fitbit group compared with the tailoring-only group. This is not consistent with the findings of Vandelanotte et al [22], who found tailored advice based on Fitbit data to be significantly more effective at increasing self-reported physical activity compared with a tailoring-only group in middle-aged adults. It is possible that older adults do not benefit as much as younger adults from tailored advice based on Fitbit data, stemming from their lower interest in accelerometer-based activity trackers [23]. Future generations of older adults may be more familiar and interested in accelerometer-based activity trackers [24].

This study did not observe a difference in self-reported physical activity or accelerometer-measured MVPA over time between the tailoring-only and control groups. Previous research has demonstrated the overall effectiveness of computer-tailored physical activity advice in middle-aged adults [16], but the evidence in older adults is mixed. It has been demonstrated by 1 study that computer-tailored physical activity advice is effective in adults aged ≥65 years [20]. However, a study conducted in older adults with chronic diseases [19] also found that computer-tailored physical activity advice was not effective at increasing objectively measured physical activity in adults aged ≥65 years. A possible reason for these differences is the time frame of the interventions. The effective tailored intervention of Van Dyck et al [20] was 5 weeks in duration, whereas the ineffective intervention of Volders et al [19] and our intervention were 4 months and 3 months, respectively, in duration. The longer time frame for the postintervention assessment of these studies may have made it harder to detect group by time effects because improvements in physical activity tend to decline over time [16]. Intervention strategies to improve maintenance of physical activity over time may improve the effectiveness of computer-tailored physical activity advice in older adults [46]. More randomized controlled trials in adults aged ≥65 years are needed to determine the effectiveness of computer-tailored physical activity advice in older adults. The social cognitive theory postulates that both social support and individual cognition (eg, self-efficacy, outcome expectancies, and intentions) are important drivers of behavior [33]. In line with this, social support is important for health behavior change in older adults [47]. The lack of social support delivered through computer-tailored physical activity programs may also partially explain their limited effectiveness in adults aged ≥65 years. Future computer-tailored interventions for older adults may need to include additional components such as advice based on activity trackers or social support components to improve effectiveness.

The increase in objectively measured sedentary behavior in the tailoring+Fitbit group is not in line with a meta-analysis that found no intervention effect on sedentary behavior outcomes for either interventions targeting physical activity alone or those targeting both physical activity and sedentary behavior [48]. However, together these findings support that interventions must focus on sedentary behavior change rather than physical activity to see improvements in sedentary behavior. Our intervention targeted physical activity; however, sedentary behavior was briefly discussed in 2 modules where participants were encouraged to reduce their sitting time if it was >8 hours a day. It is unlikely that the intervention’s focus on MVPA as well as strength, balance, and flexibility exercises caused an increase in sedentary activities because the control group participants equally increased their sedentary behavior. The discrepancy between the objectively measured and self-reported sedentary behavior outcomes may be due to the participants’ social desirability bias [49]. The improvement in self-reported sitting time at 12 weeks for the tailoring+Fitbit group compared with the control group might be because the tailoring+Fitbit group participants were more conscious about reducing sitting time, given that their sedentary behavior was being tracked. Of note, this did not translate into improvements in accelerometer-measured sedentary behavior.

### Strengths and Limitations

The strengths of this study include the 3-group randomized design to determine individual effects of a tailored web-based physical activity intervention with and without Fitbit integration compared with a wait-list control group. The study objectively assessed physical activity at baseline and after the intervention. Although attrition at week 12 was moderate (77/243, 31.7%), this is comparable to many other trials examining web-based interventions [50-52]. Although the monetary incentive for completing the research surveys is unlikely to have had a large impact, it is possible that it increased participation in the intervention itself, going by the greater engagement in the overall study. The face-to-face meetings with researchers before and after the intervention may have also increased engagement in the intervention or helped to remove barriers to participation (eg, syncing the Fitbit device to the website). Therefore, lower engagement or additional barriers to participation may arise if the intervention is administered without monetary incentives or face-to-face meetings. The lack of accelerometer-assessed MVPA data at week 24 for the main outcome measure is a limitation. As such, we do not know whether the significant difference between the tailoring+Fitbit group and the control group at 12 weeks would remain at 24 weeks. The self-reported physical activity outcomes at 24 weeks suggest that physical activity changes were maintained in all groups, but this needs to be interpreted with caution because of the large differences in objectively measured and self-reported physical activity at baseline and week 12. Participants who completed the week 12 outcomes had a lower BMI than those who dropped out. Therefore, the findings cannot be generalized to older adults with a higher BMI. Another limitation is the lack of a Fitbit-only group with participants who receive a Fitbit device to track their physical activity without also receiving any tailored advice. This would help to determine whether the improvements in the tailoring+Fitbit group were due to being tracked by the Fitbit device or the combination of the tailored advice based on the Fitbit data. The Fitbit device had 5 lights, each of them indicating an additional 2000 steps reached for the day, which may have also motivated this group to maintain their activity, independent of the computer-tailored advice. Accelerometer wear time increased between baseline and week 12. Although analyses controlled for wear time, the increase in wear time may have had some effect on the decrease in MVPA and increase in sedentary behavior observed in some groups. Furthermore, the number of participants randomized to each group varied because of small numbers recruited within some randomization groups (eg, older men) with block sizes within each randomization group being 15. The control group participants had a higher level of MVPA at baseline and therefore had more room to decrease their MVPA. This may have contributed to the between-group difference observed between the tailoring+Fitbit and control groups on MVPA changes at week 12. Finally, the conservative a priori sample size calculation (n=300) was not met; however, we recruited 243 participants, which is comparable to similar studies [9], and there was enough power to detect MVPA group differences between the tailoring+Fitbit and control groups.

In conclusion, computer-tailored advice based on Fitbit measurement of physical activity in older adults is likely to lead to improved physical activity outcomes compared with no advice but not compared with advice based on self-reported physical activity. More research is needed to investigate ways to further improve effectiveness of computer-tailored advice based on Fitbit measurement in older adults.

## Appendix

Multimedia Appendix 1 CONSORT-EHEALTH checklist (V 1.6.1).

## Abbreviations

MVPA: moderate to vigorous physical activity
